# Immune Modulatory Effects of IL-22 on Allergen-Induced Pulmonary Inflammation

**DOI:** 10.1371/journal.pone.0107454

**Published:** 2014-09-25

**Authors:** Ping Fang, Li Zhou, Yuqi Zhou, Jay K. Kolls, Tao Zheng, Zhou Zhu

**Affiliations:** 1 Respiratory Department, The Second Affiliated Hospital, Xi’an Jiaotong University School of Medicine, Xi’an, Shaanxi, China; 2 Division of Allergy and Clinical Immunology, Department of Internal Medicine, Johns Hopkins University School of Medicine, Baltimore, Maryland, United States of America; 3 Division of Pediatric Rheumatology, Children’s Hospital of Pittsburgh, University of Pittsburgh School of Medicine, Pittsburgh, Pennsylvania, United States of America; French National Centre for Scientific Research, France

## Abstract

IL-22 is a Th17/Th22 cytokine that is increased in asthma. However, recent animal studies showed controversial findings in the effects of IL-22 in allergic asthma. To determine the role of IL-22 in ovalbumin-induced allergic inflammation we generated inducible lung-specific IL-22 transgenic mice. Transgenic IL-22 expression and signaling activity in the lung were determined. Ovalbumin (OVA)-induced pulmonary inflammation, immune responses, and airway hyperresponsiveness (AHR) were examined and compared between IL-22 transgenic mice and wild type controls. Following doxycycline (Dox) induction, IL-22 protein was readily detected in the large (CC10 promoter) and small (SPC promoter) airway epithelial cells. IL-22 signaling was evidenced by phosphorylated STAT3. After OVA sensitization and challenge, compared to wild type littermates, IL-22 transgenic mice showed decreased eosinophils in the bronchoalveolar lavage (BAL), and in lung tissue, decreased mucus metaplasia in the airways, and reduced AHR. Among the cytokines and chemokines examined, IL-13 levels were reduced in the BAL fluid as well as in lymphocytes from local draining lymph nodes of IL-22 transgenic mice. No effect was seen on the levels of serum total or OVA-specific IgE or IgG. These findings indicate that IL-22 has immune modulatory effects on pulmonary inflammatory responses in allergen-induced asthma.

## Introduction

Allergen-induced pulmonary responses in asthma are characterized by eosinophil infiltration, mucus hypersecretion, airway hyperreactivity and bronchoconstriction. Th2 cytokines, IL-4 and IL-13, play a central role in orchestrating these responses, whereas Th1 cytokine IFN-γ may have opposing effects [1]–[4]. Furthermore, the Th17 cytokine IL-17A is critical in the pathogenesis of severe asthma [4], [5]. Recently, a novel Th17/Th22 cytokine, IL-22, was found to have immune modulatory effects on pulmonary allergic inflammation [6]–[8].

Th17/Th22 cells mainly secrete IL-17A, IL-17F and IL-22 [9], [10]. Both IL-17 and IL-22 have been found to have a major impact in epithelial cells in various tissues and are key regulators of homeostasis and epithelial barrier function. However, IL-22 also promotes tissue inflammation [11], [12]. Furthermore, the immunological effects of these cytokines vary in different contexts. It has been recognized that IL-17 has an important role in the recruitment of neutrophils in response to bacterial infection and a potential role in severe asthma, which may contribute to corticosteroid resistance [4], [13]. However, the immune modulatory effects of IL-22 in allergen-induced lung inflammation are not well understood.

IL-22, a member of the IL-10 family cytokines, plays critical roles in innate and adaptive immunity. In the gastrointestinal tract, innate lymphoid cells (ILCs) are a dominant source of IL-22 [7], [14]–[16]. Other cells, including CD4+ Th1, Th17, Th22 cells, CD8+ Tc17, Tc22 cells, and γδ T cells and NK cells can also produce IL-22 [17]–[21]. Interestingly, IL-22R1, a subunit of IL-22 receptor, is only found in tissue epithelial cells, such as skin, pancreas, intestine, liver, lung and kidney, which determines the tissue specificity of the biological effects of IL-22 [7], [11]. In murine lung, IL-22Ra1 is expressed in the conducting airway in both ciliated and non-ciliated cells [22]. Activation of proliferative and/or anti-apoptotic genes may be the main mechanisms mediating IL-22 immune responses. Signaling pathways, including Jak-STAT-particularly STAT3, MAPK-Akt, and bcl-2, have been found as critical downstream pathways for IL-22 functions [17], [23], [24]. IL-22 has been shown to play a key role in controlling bacteremia in experimental gram-negative pneumonia [25] and airway repair after influenza infection [22].

In clinical studies, IL-22 expression has been found to be elevated in the blood of asthmatic patients, which correlates with the disease severity [26]. Also, increased levels of IL-22 were found in the serum of asthmatic patients and in the lung tissues in experimental asthma in mice [27]. Accumulating evidence indicates that IL-22 may have immune modulatory effects on the development of allergen-induced pulmonary inflammation. However, the findings from different studies were controversial [27]–[29]. To further understand the role of IL-22 in allergic asthma, we developed inducible transgenic mice that express IL-22 specifically in the airways to investigate the immune modulatory effects of this cytokine and its underlying mechanisms in the context of OVA-induced lung inflammation.

## Materials and Methods

### Generation of inducible lung-specific IL-22 transgenic mice

IL-22 transgenic mice were generated as described previously [30] and more details are in the Supporting Information (**Figure S1 and Figure S2**). The DNA fragment containing the TRE-Tight (Clontech) promoter, IL-22 cDNA, and the SV40 polyadenylation signal was prepared and microinjected into pronuclei. TRE-Tight-IL-22 mice were identified and crossbred with the CC10-rtTA or SPC-rtTA transgenic mice [31], [32] (kind gifts from Dr. Jeffrey Whitsett, the University of Cincinnati) to produce CC10-rtTA-IL-22 or SPC-rtTA-IL-22 double Tg(+) mice. Tg(−) or wild type (WT) littermates were used as controls. In this study terms Tg(−) and WT are interchangeable. All mice were on C57BL/6 genetic background. Studies on animals were approved by the IACUC of the Johns Hopkins University.

### Induction of IL-22 expression in the lung

The IL-22 transgene was not activated until the mice were 4 weeks old. Doxycycline (Dox) was added to the animal’s drinking water (0.5 mg/ml with 4% sucrose) [33]. For all experiments, Tg(+) and WT littermates were randomized to receive normal or Dox water for 4 weeks and OVA sensitization and challenge were performed as described below.

### OVA-induced allergic asthma

Allergen sensitization and challenge were carried out as previously described [34]. Briefly, 4 weeks after Dox induction mice were divided into four groups: WT mice-PBS, WT mice-OVA, IL-22 Tg(+) mice-PBS, and Tg(+) mice-OVA. Sensitization was started by i.p. injection of 50 µg of OVA (Grade V, Sigma-Aldrich, St. Louis, MO) mixed with Alum (2 mg in 200 µl of PBS) on day 0 and day 7. The mice were challenged intranasally (i.n.) with 50 µg OVA on days 14, 15, and 16, pulmonary function tests (PFT) were performed on day 17, and sacrifice on day 18. Serum samples, bronchoalveolar lavage (BAL) fluids and cells, draining lymph nodes (DLN) and lung tissues were collected and stored until evaluation.

### Assessment of pulmonary physiology

Pulmonary function tests (PFT) were performed 24 hours after last OVA challenge [34]. Briefly, mice were anesthetized and, through a cannula, connected to FlexiVent (SCIREQ, Montreal, PQ, Canada) and mechanically ventilated. Lung resistance was measured by using the forced oscillation technique [35]. Airway hyperresponsiveness (AHR) to increasing doses of inhaled Methacholine (MCh) was determined. Data were collected at 1-minute intervals and the values for lung resistance (cm H_2_O/ml/s) were plotted as a function of MCh doses.

### Lung and bronchoalveolar lavage samples

Lung tissue and BAL samples were obtained as previously described [34], [35]. Briefly, mice were anesthetized and a small-caliber tubing was inserted into the trachea. Two successive volumes of 1 ml of PBS were instilled, aspirated and pooled. BAL samples were centrifuged at 4000 rpm, and supernatants were stored at −80°C until evaluation. Cells in 100 µl aliquots were counted by trypan blue staining. A total of 100,000 viable BAL cells were centrifuged in Cytospin 3 (Thermo Shandon Ltd, Runcorn, UK). Cell differentiation was determined by Diff-quik (Fisher Scientific Co., Newark, DE). The lung was perfused with cold PBS. The whole lung was either excised for RNA and protein analyses or inflated with neutral buffered formalin for histology.

### Histology, immunohistochemistry (IHC), and immunofluorescence

H&E and Alcian blue (AB) stains were performed on lung sections as described [35]. For IHC, monoclonal antibody against eosinophil specific major basic protein (anti-MBP, a kind gift from Drs. Nancy and James J. Lee, Mayo Clinic, Scottsdale, AZ) was used (1∶500) for eosinophils and anti-IL-22 antibody was used to detect IL-22 (1∶180). ABC staining kits were used to amplify the signal (Santa Cruz Biotechnology). Immunofluorescence of p-STAT3 was performed with rabbit anti-mouse phospho-Stat3 (Tyr705) (Cell Signaling, Danvers, MA) and Alexa Fluor 488-labeled donkey anti-rabbit IgG as secondary antibody (Invitrogen) and DAPI for nuclei. Tissue sections were mounted and examined using Zeiss LSM 510 laser scanning confocal microscope (Carl Zeiss) at 350 nm to assess p-STAT3 and 405 nm to assess cell nuclei.

### Analysis of mRNA

Total cellular RNA from lung tissue was collected and analyzed by RT-PCR using specific primers for IL-22 and β-actin as detailed in Supporting Information (**Figure S1 and Figure S2**).

### Cytokine production by lymphocytes from draining lymph nodes (DLN) and spleen

Lymphocytes from DLN (mediastinal and hilar) and purified splenic CD4+ cells were cultured in RPMI1640 medium (5% FCS) and were stimulated with medium as control, OVA (50 µg/ml) for 5 days or anti-CD3/CD28 (BD Bioscience) (5 µg/ml) for 3 days. Supernatants were collected for cytokine measurement.

### Quantification of cytokines, chemokines, and immunoglobulins

Cytokines and chemokines in the BAL or supernatant from lymphocytes were determined using commercially available ELISA kits according to the manufacturer’s instructions (R&D Systems, Minneapolis, MN; eBioscience, San Diego, CA). Serum samples in duplicates were analyzed using ELISA kits for total and specific IgE, IgG1, and IgG2a (BD Biosciences, San Jose, CA) per manufacturer’s instructions.

### Western blot

Rabbit anti-mouse STAT3 and phospho-STAT3 (Tyr705) monoclonal antibodies (Cell Signaling, Fremont, CA) were used as primary Abs (Neomarker). An anti-rabbit IgG conjugated to HRP was used as secondary Ab (Santa Cruz Biotechnology). Protein detection was accomplished with a Super Signal West Femto Maximum Kit (Pierce).

### Statistical analysis

The Student t-test was used to determine the significance of difference between two groups, and one-way ANOVA was used for comparison among multiple groups. Data were expressed as Mean±SEM and differences with *P* values = <0.05 were considered statistically significant.

## Results

### Generation of lung-specific inducible IL-22 transgenic mice

**Figure 1:**
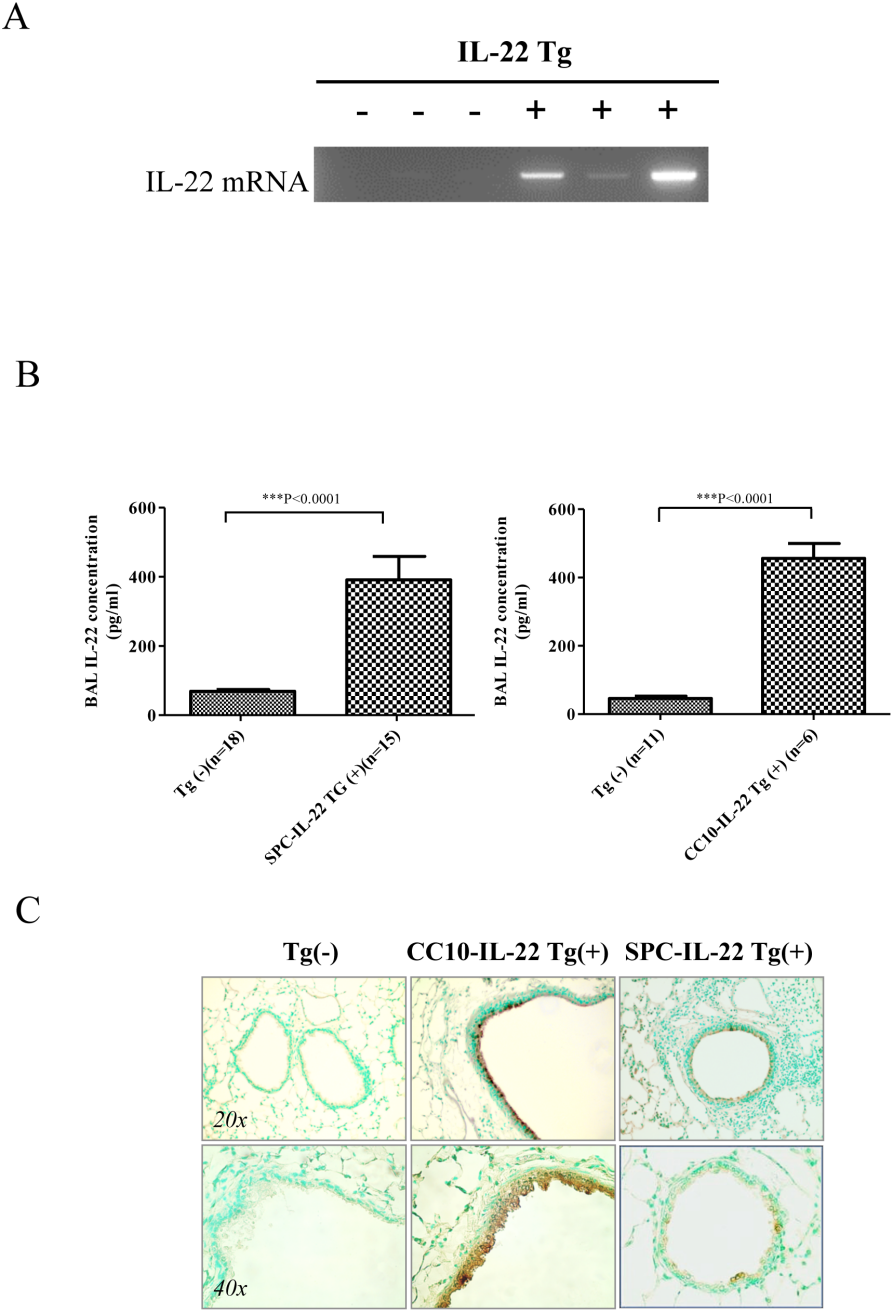
Targeted IL-22 expression in the airway epithelial cells. (A) IL-22 mRNA expression in the lung of Tg(+) mice after being given doxycycline (Dox) water for 4 weeks compared to Tg(−) mice. (B) IL-22 protein in the BAL samples expressed by airway epithelial cell was measured by ELISA. Both SPC-IL-22 and CC10-IL-22 Tg(+) mice showed much higher levels of IL-22 compared to Tg(−) mice (*P*<0.0001). (C) Identification of localization of IL-22 expression by IHC in the airway epithelial cells. In CC10-IL-22 mice IL-22 was mainly expressed in the large airways, whereas in SPC-IL-22 mice, mainly in the small airways.

Studies show that transgenic mice with liver overexpression of IL-22 survived but lymphoid lineage-specific overexpression of IL-22 caused neonatal mortality [36], [37]. To avoid potential lethality, an inducible transgenic approach was taken to overexpress IL-22 in the lung. As illustrated in **Figure S1**, we generated mice carrying transgenes CC10-rtTA or SPC-rtTA and TRE-Tight-IL-22 (CC10-rtTA-IL-22) or (SPC-rtTA-IL-22) and these mice were used in the OVA-induced asthma experiments. The CC10 and SPC promoter controlled rtTA is activated only in the presence of Dox. To express IL-22 only in the adult mice, Dox water was not given to Tg(+) mice and Tg(−) littermates until they were 4 weeks of age (**Figure S2**). Inducible tissue-specific expression of IL-22 in the airways was tested after 4 weeks of Dox and the induction was kept on for the entire experiment. Without Dox water, IL-22 was not detected either at the mRNA or protein level in any tissue of Tg(+) and Tg(−) mice (**Figures 1A–C**). However, after Dox induction for 4 weeks, IL-22 mRNA was readily detected in the lung tissue and IL-22 protein was elevated in the bronchoalveolar lavage (BAL) fluid of Tg(+) mice but not in Tg(−) mice by ELISA (**Figure 1A, 1B**). Immunohistochemistry (IHC) using anti-IL-22 was performed to confirm that the location of IL-22 expression was in the large airways in CC10-rtTA-IL-22 mice and in the small airways in SPC-rtTA-IL-22 mice (**Figure 1C**). These results demonstrated that using the CC10-rtTA or SPC-rtTA system, the IL-22 transgene was targeted specifically in the airways in an inducible fashion.

### Activation of STAT3 by IL-22 expressed in the lung

**Figure 2:**
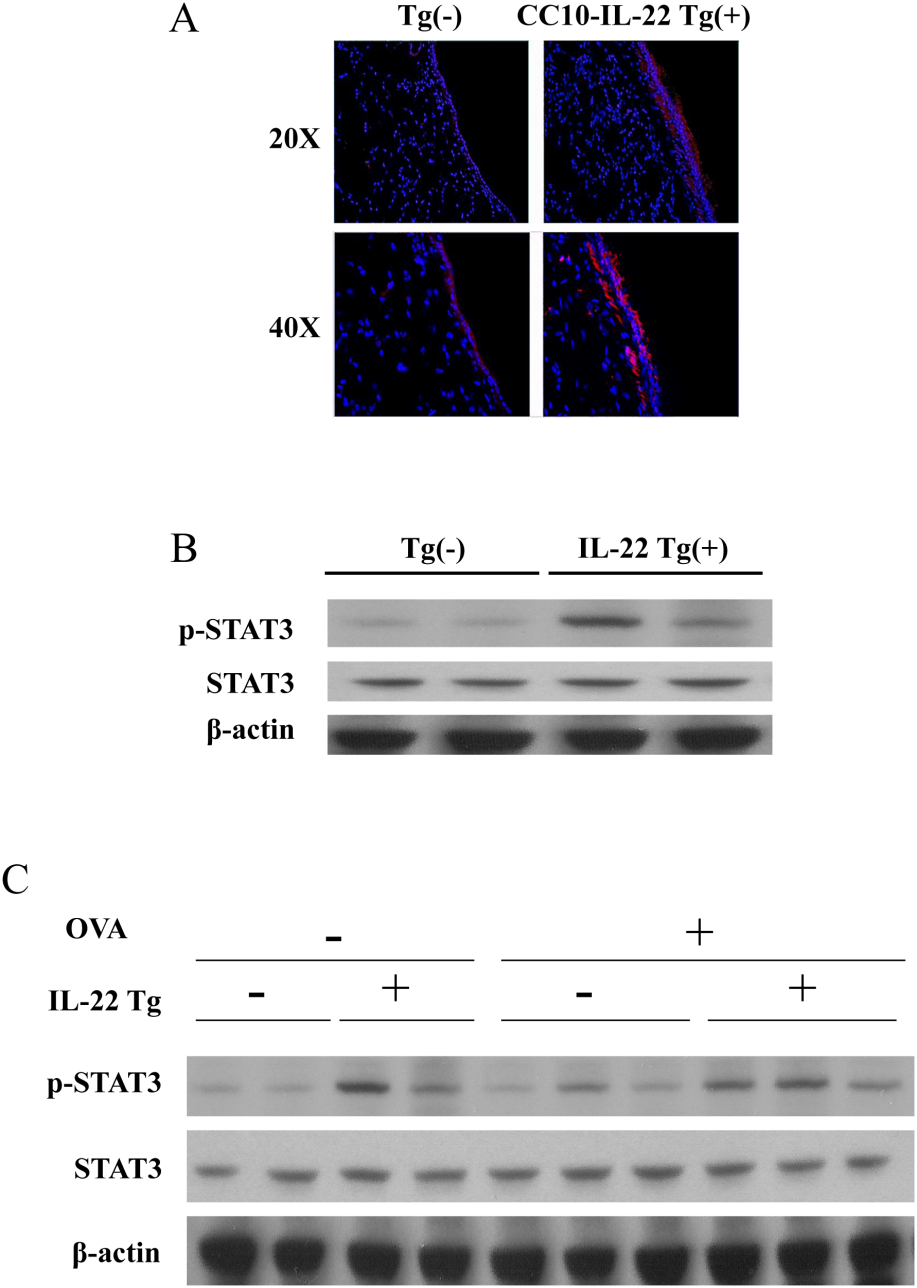
Activation of p-STAT3 by IL-22 in the lung of Tg(+) mice. (A) Phospho-STAT3 protein expression activated by IL-22 in the airway epithelial cells of IL-22 Tg(+) mice and compared to background level in Tg(−) mice using immunofluorescence (IF). (B) Phospho -STAT3 protein expression in the lung tissue of IL-22 Tg(+) and Tg(−) mice detected by Western blot using anti-p-STAT3 antibody. (C) Phospho-STAT3 protein expression in the lung tissues of OVA-stimulated IL-22 Tg(+) and Tg(−) mice detected by Western blot.

To determine whether IL-22 expressed in the airways was biologically active, activation of its downstream signaling molecule STAT3 was tested. Using immunofluorescence (IF), we demonstrated that inducible IL-22 activated p-STAT3 in the airways of Tg(+) mice compared to background staining in Tg(−) mice (**Figure 2A**). By Western blot, p-STAT3 was found to be increased in the lung tissues of Tg(+) mice compared to Tg(−) mice at baseline (with PBS) (**Figure 2B**). OVA sensitization and challenge induced increased p-STAT3 above baseline in Tg(−) mice but p-STAT3 was further increased in Tg(+) mice (**Figure 2C**). These results indicate that inducible IL-22 expression activated its signaling pathway in the airway epithelial cells and OVA stimulation further increased p-STAT3.

### Protective effect of IL-22 on allergic airway inflammation

**Figure 3:**
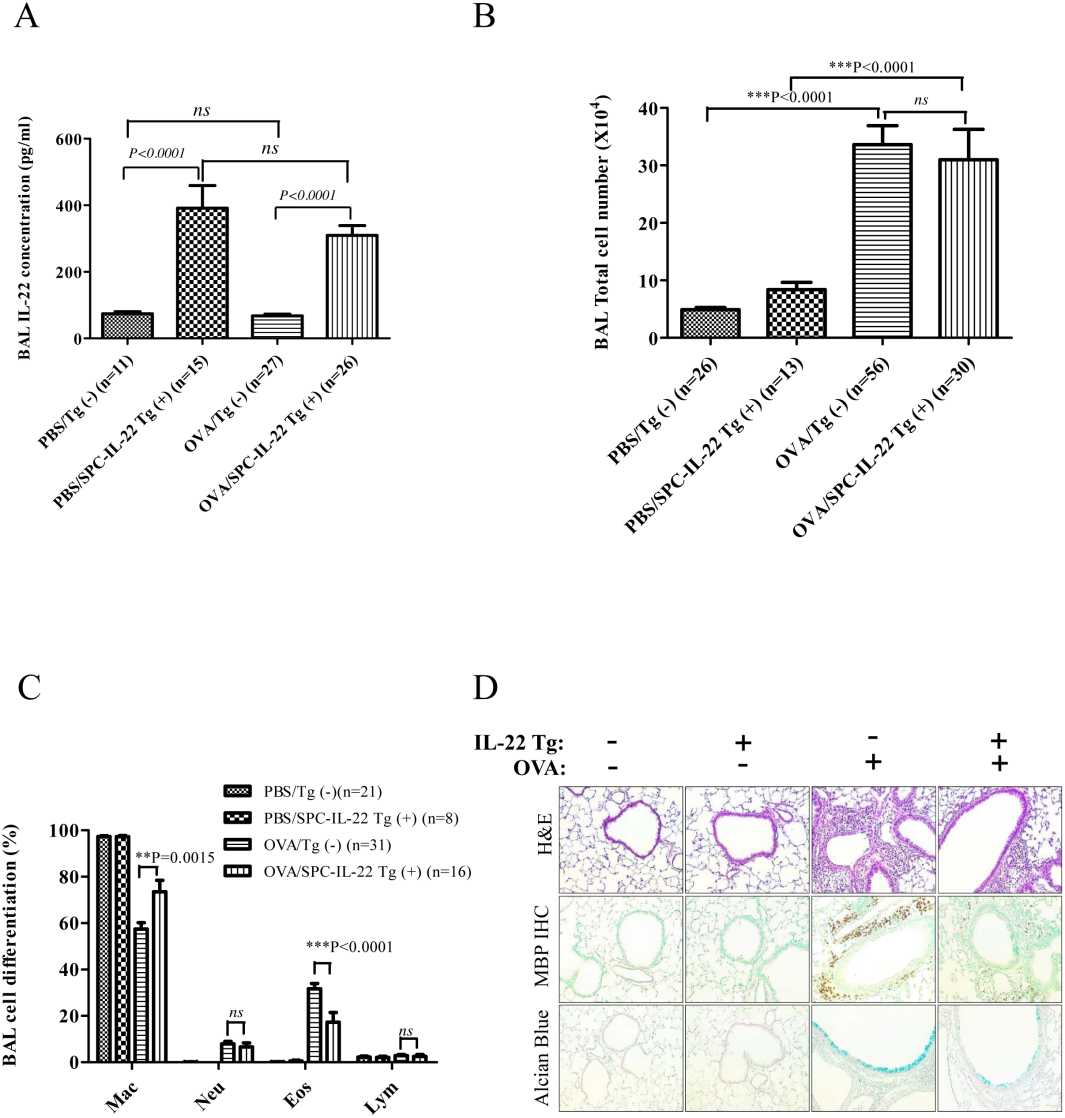
IL-22 alleviated OVA-induced eosinophilic inflammation in the lung. (A) High levels of IL-22 cytokine were seen in the BAL of PBS and OVA-stimulated IL-22 Tg(+) mice without difference between the two groups (*P*>0.05). When compared to Tg(−) mice, IL-22 concentrations in the BAL of Tg(+) mice were much higher than that in PBS and OVA-stimulated control groups (*P*<0.0001). (B, C) BAL total cell and differentials counts showed that OVA-stimulated IL-22 Tg(+) group had a much higher percentage of eosinophils compared to OVA-stimulated Tg(−) mice (*P*<0.0001), but there is no difference in the total cell counts (*P*>0.05). (D) Lung histology of OVA-induced allergic asthma in SPC-IL-22 Tg(+) mice and Tg(−) mice, H&E, IHC for MBP, and Alcian blue staining showed that OVA-induced IL-22 Tg(−) group had much more severe airway inflammation compared to OVA-induced IL-22 Tg(+) group.

To verify directly whether IL-22 has any modulatory effect on allergen induced lung inflammation, Tg(−) and IL-22 Tg(+) mice were sensitized on days 0 and 7 with OVA, followed by three OVA challenges through the airway on days 14, 15 and 16. In PBS control groups, Tg(−) and IL-22 Tg(+) mice showed no airway inflammation or eosinophilia. Similar to what we had observed in non-allergen challenged mice, IL-22 levels in the BAL were elevated in IL-22 Tg(+) mice (**Figure 3A**). After OVA sensitization and challenge, both Tg(−) and Tg(+) groups showed significantly increased BAL cells compared to PBS challenged control groups. However, no difference was noticed in the total BAL cell numbers between the two Tg(−) and Tg(+) OVA groups (**Figure 3B**). In cell differential counts, OVA stimulated Tg(−) group (OVA/Tg(−)) showed substantial eosinophilic airway inflammation, while the number of eosinophils in the BAL of OVA treated IL-22 Tg(+) (OVA/Tg(+)) group was significantly less compared to the OVA/Tg(−) group (**Figure 3C**). Eosinophil infiltration in the lung tissue (H&E) also showed a reduction in the OVA/Tg(+) group compared to the OVA/Tg(−) group (**Figure 3D**). IHC analysis of eosinophil-specific MBP in the tissue confirmed the reduction of eosinophils in the lung tissue of OVA/Tg(+) mice vs. OVA/Tg(−) mice (**Figure 3D**). Mucus metaplasia is another important feature of allergic asthma. Analysis using Alcian Blue staining of lung sections showed that IL-22 Tg(+) mice had less mucus producing cells in the airways than Tg(−) mice after OVA stimulation (**Figure 3D**). CC10-IL-22 mice showed similar results (data not shown). These results demonstrated that IL-22 has protective effects on OVA induced allergic airway inflammation (**Figure 3**).

### IL-22 alleviates OVA induced airway hyperresponsiveness

**Figure 4:**
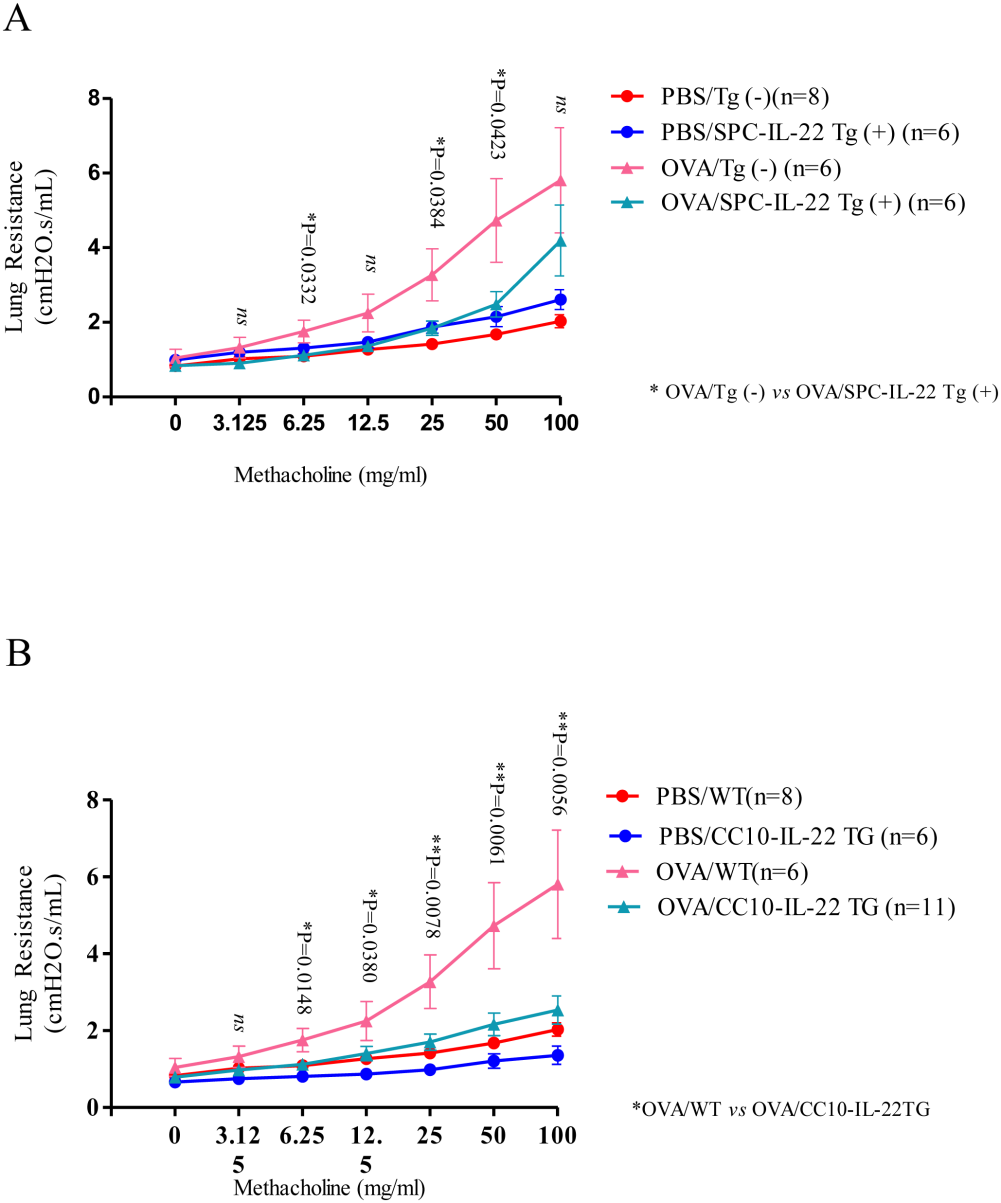
IL-22 attenuated airway hyperresponsiveness (AHR). (A, B) Invasive PFT of OVA stimulated IL-22 Tg(+) and Tg(−) mice was assessed (FlexiVent, SciQuest). Lung resistance at baseline and in response to increasing concentrations of methacholine (MCh) through inhalation was recorded and analyzed (*P<0.05). The number of animals used in each group was as indicated. Data represented as Mean±SEM. OVA-induced IL-22 Tg(+) mice showed significantly lower lung resistance compared to OVA-induced IL-22 Tg(−) mice.

Analysis of the lung mechanics using the forced oscillation technique (FlexiVent) demonstrated that, in the absence of OVA at baseline, IL-22 Tg(+) mice did not show any significant difference in lung resistance compared to that of Tg(−) mice (**Figure 4**). After OVA challenge, as expected, in response to increasing doses of MCh, Tg(−) mice showed large increments in lung resistance above baseline. However, IL-22 Tg(+) mice that received OVA showed significantly reduced lung resistance at MCh concentrations above 6.25 mg/ml. Similar results were seen in CC10-rtTA-IL-22 and SPC-rtTA-IL-22 mice (**Figure 4A, 4B**). These data indicate that IL-22 in the airways protects mice from allergen induced airway hyperresponsiveness.

### Lung-specific expression of IL-22 did not affect OVA-induced immunoglobulin responses

**Figure 5:**
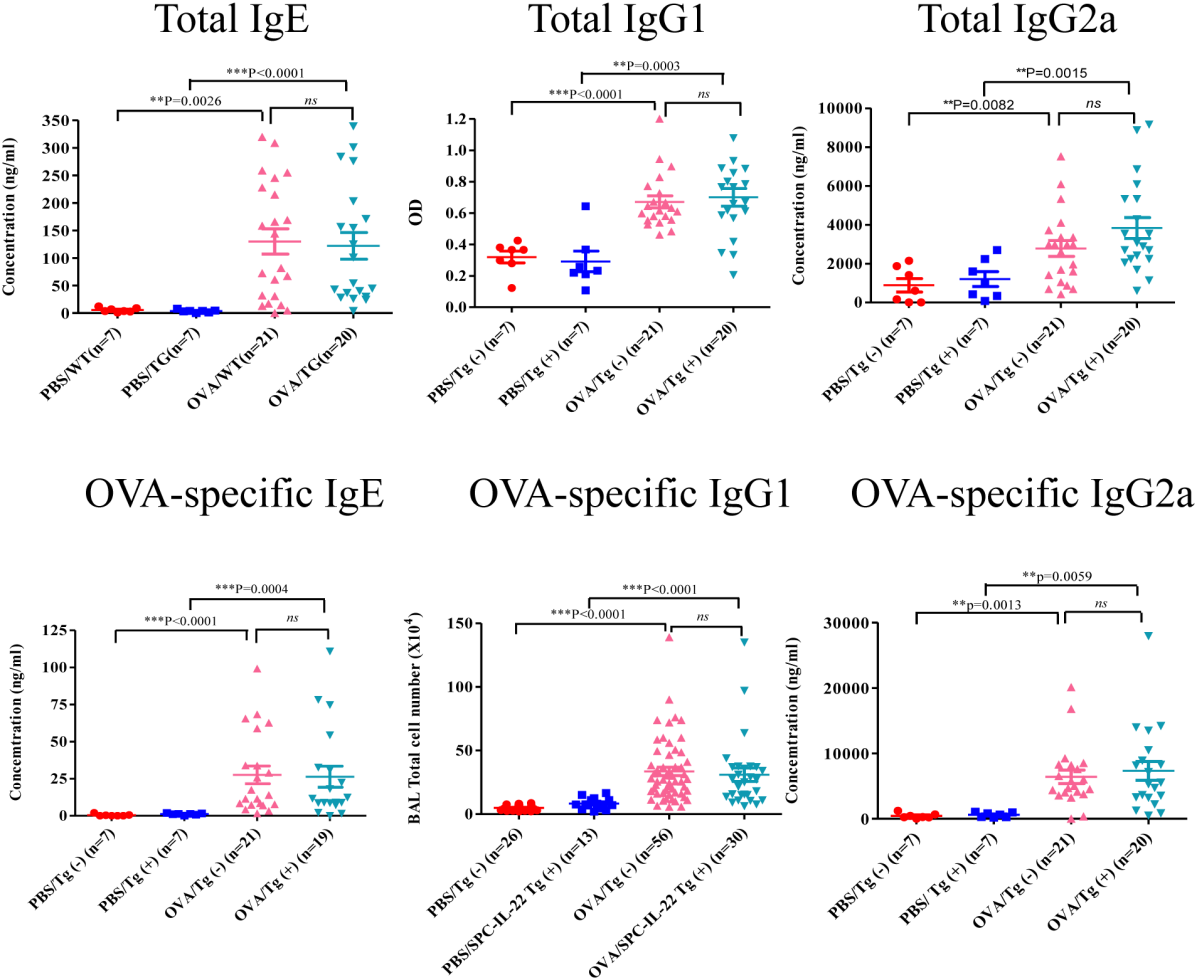
Serum immunoglobulin levels in OVA-induced allergic asthma in IL-22 Tg(+) (SPC-IL-22) and Tg(−) mice. Serum samples from IL-22 Tg(+) and Tg(−) mice were collected 48 hours after last OVA challenge. Immunoglobulins, including total and OVA-specific IgE, IgG1 and IgG2a were measured by ELISA and analyzed by one-way ANOVA. Data from individual animals were plotted. Both IL-22 Tg(+) and Tg(−) group showed much higher level either in total or in OVA-specific IgE, IgG1 and IgG2a than PBS treatment groups (*P*<0.01). But there is no difference between IL-22 Tg(+) and Tg(−) groups (*P*>0.05).

To determine whether inducible IL-22 expression in the lung has any effect on the systemic immune response in allergen-induced inflammation, serum samples were obtained and total and OVA-specific immunoglobulins, IgE, IgG1 and IgG2a, in the serum were measured by ELISA. Mice received PBS had only baseline total immunoglobulins in the serum without any allergen-specific IgE or IgGs. OVA induced elevated total and OVA-specific IgE, IgG1 and IgG2a in the serum of Tg(−) and SPC-IL-22 Tg(+) mice (**Figure 5**). However, no significant difference was noted between IL-22 Tg(+) and Tg(−) mice that received OVA. CC10-IL-22 mice showed similar results (data not shown). These results indicate that inducible IL-22 expression in the airway epithelial cells had no effect on immunoglobulin responses.

### Effect of IL-22 on cytokine and chemokine expression in the lung

**Figure 6:**
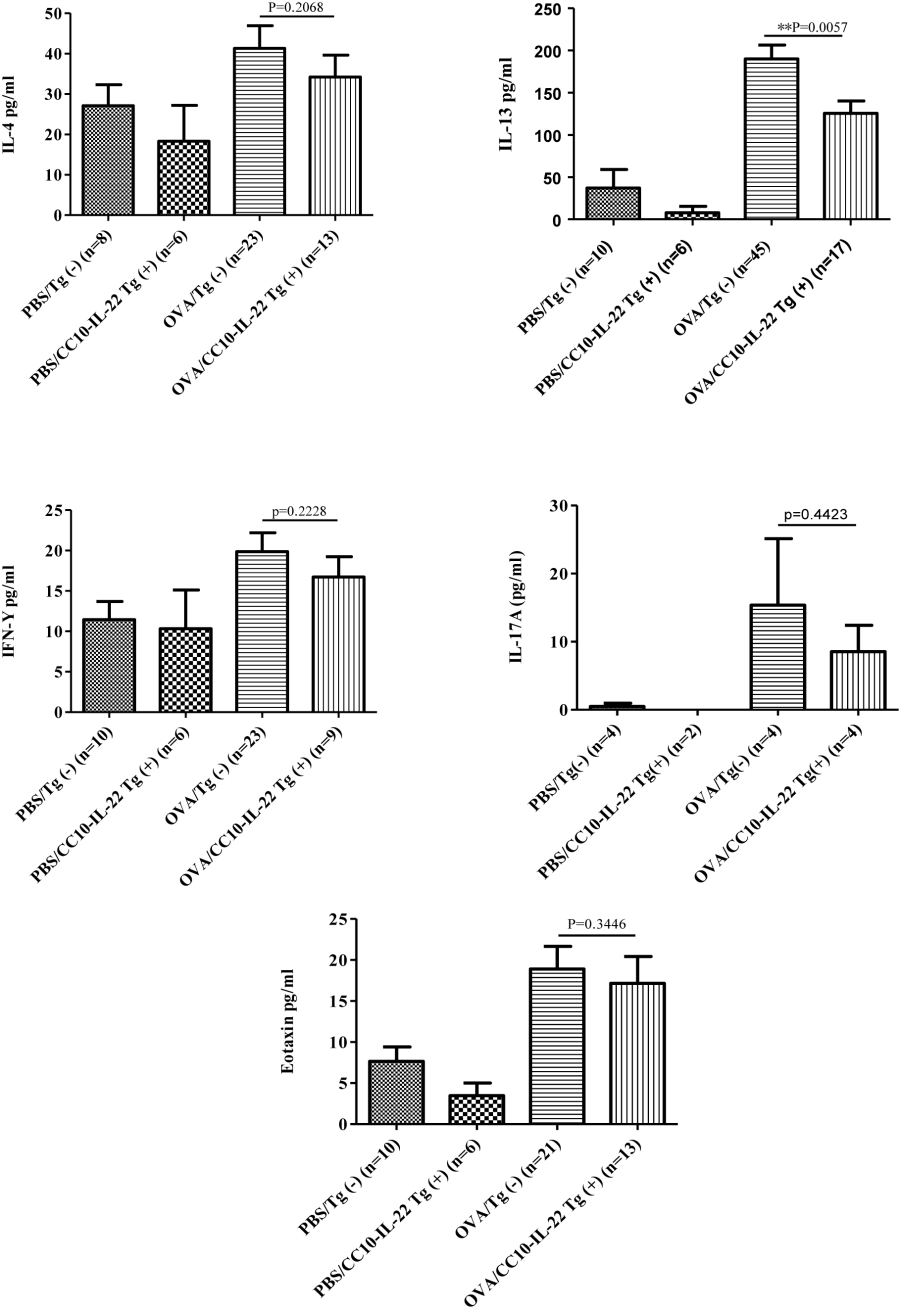
Effect of IL-22 (CC10-IL-22) on cytokine and chemokine production in OVA-induced allergic asthma. Th1 cytokine, IFN-γ, and Th2 cytokines, IL-4 and IL-13, Th17 cytokine IL-17A, and chemokine eotaxin in the BAL were measured by ELISA. The number of animals in each group was indicated and data were shown as Mean±SEM. **P<0.01 (unpaired Student t-test).

We next examined whether the inhibitory effect of IL-22 on OVA-induced allergic airway inflammation was through regulation of inflammatory mediators, such as cytokines and chemokines that control cell migration. BAL samples were collected after OVA challenge for cytokine and chemokine measurement. Overall, OVA stimulation induced elevated levels of cytokines and chemokines above the baseline in PBS groups. Compared to OVA/Tg(−) mice, OVA/CC10-IL-22 Tg(+) mice showed significantly lower levels of IL-13, but not IL-4, in the BAL. Th1 cytokine, IFN-γ, showed a slight increase in OVA treated groups compared to the PBS groups, but no statistical difference was noted between Tg(−) and Tg(+) mice. There was also a trend of reduction in IL-17A and eotaxin in Tg(+) mice, but the difference between Tg(+) and Tg(−) mice was not statistically significant (**Figure 6**). SPC-IL-22 mice showed similar results (data not shown).

### IL-22 inhibition of cytokine production by local lymphocytes, but not splenocytes

**Figure 7:**
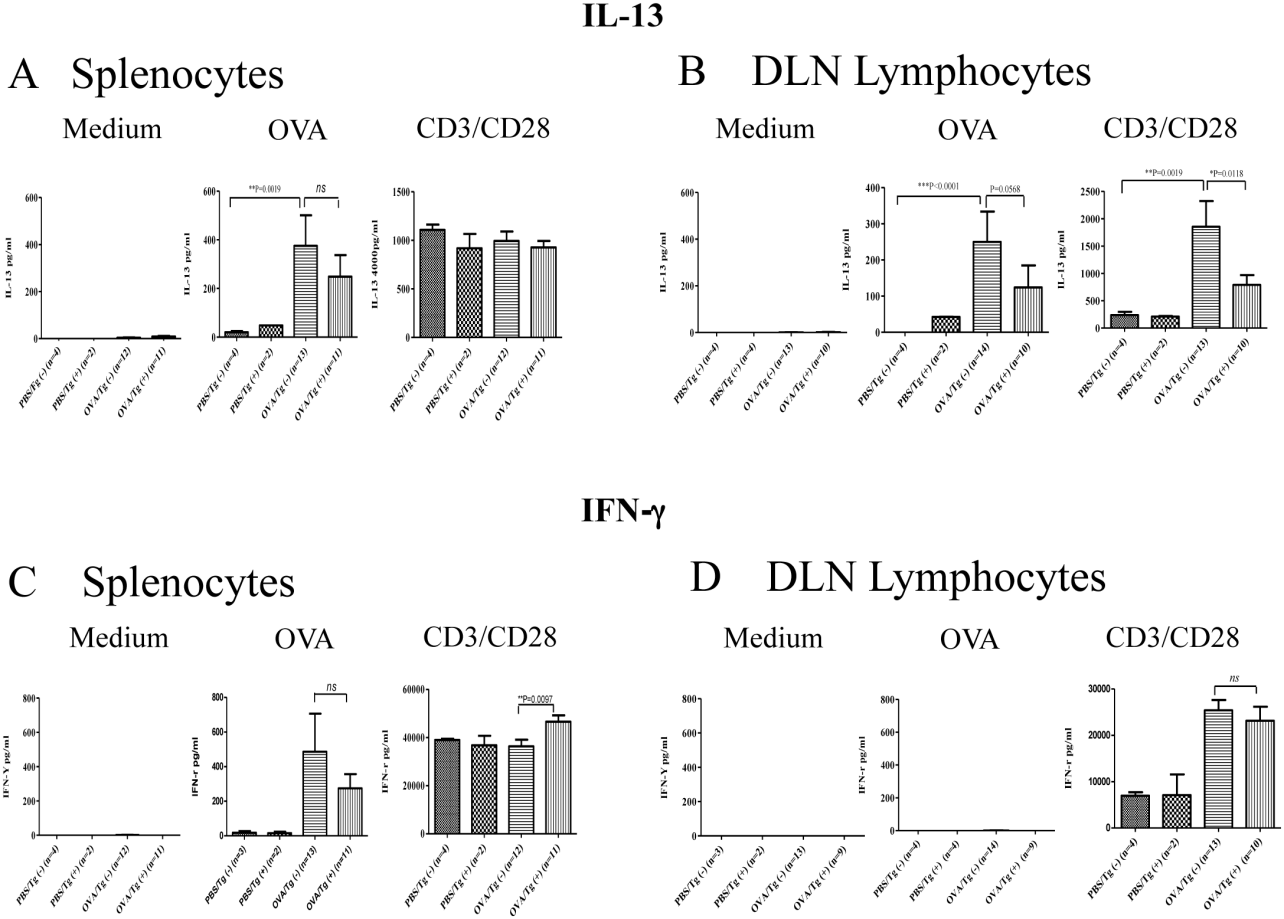
Effect of IL-22 (SPC-IL-22) on OVA-induced systemic and local immune responses. Splenocytes and lymphocytes from peribronchial draining lymph nodes (DLN) from IL-22 Tg(+) and Tg(−) mice after OVA challenge were cultured and stimulated with medium control, OVA or CD3/CD28. Th1 cytokine, IFN-γ, and Th2 cytokine, IL-13 in the supernatant were measured by ELISA. The number of animals used in the experiments was indicated and data were shown as Mean±SEM. *P<0.05 and **P<0.01 (unpaired Student t-test).

To determine if IL-22 inhibition of airway allergic inflammation was associated with systemic or local immune responses, splenocytes and lymphocytes from DLN of Tg(+) and Tg(−) mice after OVA stimulation were isolated and cultured. After stimulation with OVA or anti-CD3/CD28 supernatant was collected and cytokines IL-13 and IFN-γ were measured by ELISA. At baseline, cells cultured in media alone did not show any cytokine. We also did not observe cytokine responses in immune cells from PBS groups in response to OVA stimulation. OVA sensitized splenocytes and DLN lymphocytes from Tg(−) mice showed robust responses to antigen and TCR stimulation by producing IL-13 and IFN-γ. However, DLN lymphocytes, but not splenocytes, from IL-22 Tg(+) mice produced significantly lower levels of IL-13 compared to cells from Tg(−) mice. (**Figure 7A, 7B**). There was no difference between cells from Tg(+) and Tg(−) mice in IFN-γ production in response to OVA or anti-CD3/CD28 (**Figure 7C, 7D**). CC10-IL-22 mice showed similar results (data not shown). These results showed that IL-22 alleviated airway allergic inflammation possibly through inhibition of the local Th2 immune response.

## Discussion

Allergic asthma is a Th2 dominant inflammatory disease in the airways. In recent years, another T cell subtype, Th17/Th22, has been added to this paradigm. Recent studies suggested that Th17/Th22 cytokines IL-17 and IL-22 have regulatory effects on allergic airway inflammation. Besides its proinflammatory effects in autoimmune diseases and host defense, IL-17 can recruit neutrophils into the airways in allergic asthma [38]. After IL-22 was first described [39], extensive studies have shown that IL-22 has immunological effects, predominantly proinflammatory, in various diseases or models, such as bleomycin induced lung inflammation, and other disease models, including arthritis, hepatitis, psoriasis, atopic dermatitis, and inflammatory bowel disease [11], [40]–[45]. In the lung, IL-22 plays a key role in controlling bacteremia in experimental gram-negative pneumonia and airway tissue repair after influenza infection [22], [25]. Currently, Phase I and Phase II clinical trials based on anti-IL-22 therapies are on going for atopic dermatitis, psoriasis, and rheumatoid arthritis. However, IL-22 related therapies for the treatment of allergic asthma have not been initiated. One possible reason is that the role of IL-22 in allergic asthma is not well defined. This is at least in part due to a lack of studies that have focused on the immunological effects of IL-22 on allergic airway inflammation and the findings from several studies are inconsistent. Some evidence suggested that IL-22 acts as a proinflammatory cytokine like IL-17, but others pointed to the opposite direction, in that IL-22 may be protective in allergic airway inflammation. The discrepancies may be caused by differences in the model systems and approaches used in these studies. For example, administration of recombinant murine IL-22, use of neutralizing IL-22 antibody or IL-22 plasmid DNA during sensitization or challenge phase as well as IL-22 knockout mice were used in allergen induced allergic asthma models. However, no study has been performed to show the direct tissue effects of IL-22 in the lung and whether IL-22 expressed in the airways has regulatory effects on allergen induced asthma. To further understand IL-22 in allergic asthma, we generated transgenic mice that express the cytokine specifically in the airway epithelial cells and investigated the immune modulating effects of IL-22 in OVA allergen induced allergic asthma.

Using the tetracycline inducible transgenic system, we established IL-22 mouse models with two different promoters, SPC and CC10, to target the expression of this cytokine in the small and large airways, respectively. The location and quantification of IL-22 expression were determined by IHC and ELISA. Without Dox induction, no IL-22 was detected in the airways. But when induced by Dox, IL-22 protein was readily detectable in the airways and in the lung tissue.

IL-22 signals through the Jak-STAT pathway, phosphorylating and activating kinases Jak1 and Tyk2 and downstream transcription factors STAT1, STAT3 and STAT5. Indeed, when IL-22 transgene was turned on by Dox, p-STAT3 was detected by IHC and Western blot in the airways of transgene positive mice, indicating that IL-22 is functional in the lung epithelial cells. In addition, OVA stimulation induced p-STAT3 in the airways in both WT and IL-22 Tg(+) mice but higher levels of p-STAT3 were maintained in the Tg(+) mice throughout the experiments.

In the present study, OVA sensitization and challenge did not induce detectable endogenous IL-22 expression in the airway of the mice. This finding is consistent with one report [4] but different from another, in which IL-22 expression was found increased in the OVA model [27]. However, in the latter study IL-22 was detected in the supernatant of PMA and ionomycin stimulated mononuclear cells isolated from the lungs of OVA treated mice. No direct detection of IL-22 in the BAL or lung tissue was reported.

In this study IL-22 expression was activated by Dox for 4 weeks before OVA sensitization and the transgene was kept on for the rest of the experiment. Thus, the IL-22 cytokine was present in the lung throughout OVA sensitization and challenge. IL-22 transgenic mice showed decreased eosinophils in the BAL and a significant reduction in eosinophilic inflammation in the lung, decreased mucus metaplasia in the airways and functionally, reduced airway hyperresponsiveness. These results were similar to those in studies by two other groups [27], [46]. They used IL-22 knockout mice and Balb/c mice to establish OVA-induced allergic airway inflammation and gave different treatment during different phases [27], [46]. IL-22 is produced by many immune cells. However, the expression of functional IL-22 receptor is restricted to nonhematopoietic tissue cells in the skin, pancreas, intestine, liver, lung and kidney. It’s known that IL-22 does not induce immunoglobulin production by human B cells [47]. In our study, the IL-22 transgene was targeted specifically in the lung. Measurement of serum immunoglobulins showed no difference between IL-22 Tg(+) mice and WT mice after OVA stimulation, indicating that IL-22 in the lung has no effect on immunoglobulin responses. This finding is different from a previous report in which OVA-specific IgE was decreased when recombinant IL-22 was given at the time of OVA challenge [27]. The reason for this discrepancy is unclear. It could be due to a much higher dose of IL-22 used in that study (1 µg/mouse, 3x), whereas the transgenic expressed IL-22 in our system was probably lower, even though IL-22 was present continuously.

Measurement of the cytokines and chemokines in the lung showed a significant reduction in IL-13 and a slight decrease in IL-4, eotaxin and a trend of reduction in IL-17A, but not IFN-γ, in IL-22 transgenic mice, suggesting that IL-22 down-regulates T-cell priming in the lung. Interestingly, however, IL-22 did not affect the generation of OVA-specific antibodies suggesting that IL-22 did not impact germinal center reactions. Notably, when looking into the modulatory function of IL-22 on the immune cells, we found no difference between WT and IL-22 mice in splenocyte production of IFN-γ, IL-4 and IL-13 in response to OVA allergen or CD3/CD28 TCR stimulation. In lymphocyte cytokine production, however, a significant reduction in IL-13 was observed, but not other cytokines, by cells from IL-22 transgenic mice. Together with the IgE data, these results indicate that IL-22 in the lung suppressed immune cell production of IL-13 and Th2 cytokines and chemokines in the lung without any effect on the systemic immune responses.

Taking the transgenic approach, we sought to understand the functional role of IL-22 in the lung in the context of allergen-induced asthma. IL-22 was continuously and consistently expressed in the small and large airways in mice. IL-22 was able to activate downstream signaling molecule STAT3 without inducing any discernable pathological changes in the lung. However, in the context of allergen-induced asthma, IL-22 suppressed eosinophilic inflammation, mucus hyperplasia, AHR, and Th2 cytokine and chemokine production without affecting the systemic immune responses. With this feature IL-22 may be considered as an immune modulator in allergic asthma. Further studies are required to elucidate the underlying mechanisms for the effects of IL-22 in the lung.

## Supporting Information

Figure S1 **Schematic DNA construct of TRE-Tight-IL-22 transgene.** IL-22 cDNA was inserted into the multiple cloning site (MCS) of pTRE-Tight vector (Clontech) using restriction enzymes and microinjected into fertilized eggs as described.
(TIF)

Figure S2 **Generation of SPC- or CC10-rtTA-TRE-Tight-IL-22 (also called SPC- or CC10-IL-22) mice.** As illustrated, SPC-rtTA or CC10-rtTA mice were crossbred with TRE-Tight-IL-22 mice to obtain SPC- or CC10-IL-22 double positive mice. The IL-22 transgene was activated by doxycycline (Dox) in the drinking water for 4 weeks. ELISA, Western blot, immunohistochemistry (IHC) and immunofluorescence (IF) were performed to identify the expression of IL-22 in the lung. Without Dox, no IL-22 was detected in the BAL or in the lung.
(TIF)

File S1 (DOCX)

## References

[1] Wong CK, Ho CY, Ko FW, Chan CH, Ho AS, et al (2001) Proinflammatory cytokines (IL-17, IL-6, IL-18 and IL-12) and Th cytokines (IFN-gamma, IL-4, IL-10 and IL-13) in patients with allergic asthma. Clin Exp Immunol 125: 177–183. 11529906 10.1046/j.1365-2249.2001.01602.x PMC1906135

[2] Lee YC, Lee KH, Lee HB, Rhee YK (2001) Serum levels of interleukins (IL)-4, IL-5, IL-13, and interferon-gamma in acute asthma. J Asthma 38: 665–671. 11758895 10.1081/jas-100107544

[3] Stirling RG, Chung KF (2000) Future treatments of allergic diseases and asthma. Br Med Bull 56: 1037–1053. 11359636 10.1258/0007142001903526

[4] Zhao J, Lloyd CM, Noble A (2013) Th17 responses in chronic allergic airway inflammation abrogate regulatory T-cell-mediated tolerance and contribute to airway remodeling. Mucosal Immunol 6: 335–346. 22892938 10.1038/mi.2012.76 PMC4233308

[5] McKinley L, Alcorn JF, Peterson A, Dupont RB, Kapadia S, et al (2008) TH17 cells mediate steroid-resistant airway inflammation and airway hyperresponsiveness in mice. J Immunol 181: 4089–4097. 18768865 10.4049/jimmunol.181.6.4089 PMC3638757

[6] Cannon MJ, Goyne H, Stone PJ, Chiriva-Internati M (2011) Dendritic cell vaccination against ovarian cancer–tipping the Treg/TH17 balance to therapeutic advantage? Expert Opin Biol Ther 11: 441–445. 21271951 10.1517/14712598.2011.554812 PMC3070487

[7] Souwer Y, Szegedi K, Kapsenberg ML, de Jong EC (2010) IL-17 and IL-22 in atopic allergic disease. Curr Opin Immunol 22: 821–826. 21087848 10.1016/j.coi.2010.10.013

[8] Hirose K, Takahashi K, Nakajima H (2013) Roles of IL-22 in Allergic Airway Inflammation. J Allergy (Cairo) 2013: 260518. 23577040 10.1155/2013/260518 PMC3594983

[9] Middleton GW, Annels NE, Pandha HS (2012) Are we ready to start studies of Th17 cell manipulation as a therapy for cancer? Cancer Immunol Immunother 61: 1–7. 22086162 10.1007/s00262-011-1151-y PMC11029090

[10] Atarashi K, Tanoue T, Umesaki Y, Honda K (2010) Regulation of Th17 cell differentiation by intestinal commensal bacteria. Benef Microbes 1: 327–334. 21831771 10.3920/BM2010.0026

[11] Sonnenberg GF, Nair MG, Kirn TJ, Zaph C, Fouser LA, et al (2010) Pathological versus protective functions of IL-22 in airway inflammation are regulated by IL-17A. J Exp Med 207: 1293–1305. 20498020 10.1084/jem.20092054 PMC2882840

[12] Akdis M, Palomares O, van de Veen W, van Splunter M, Akdis CA (2012) TH17 and TH22 cells: a confusion of antimicrobial response with tissue inflammation versus protection. J Allergy Clin Immunol 129: 1438–1449; quiz1450–1431. 10.1016/j.jaci.2012.05.003 22657405

[13] Chang Y, Al-Alwan L, Risse PA, Halayko AJ, Martin JG, et al (2012) Th17-associated cytokines promote human airway smooth muscle cell proliferation. FASEB J 26: 5152–5160. 22898922 10.1096/fj.12-208033

[14] Takatori H, Kanno Y, Watford WT, Tato CM, Weiss G, et al (2009) Lymphoid tissue inducer-like cells are an innate source of IL-17 and IL-22. J Exp Med 206: 35–41. 19114665 10.1084/jem.20072713 PMC2626689

[15] Mjosberg J, Spits H (2012) Type 2 innate lymphoid cells-new members of the “type 2 franchise” that mediate allergic airway inflammation. Eur J Immunol 42: 1093–1096. 22539283 10.1002/eji.201242549

[16] Scanlon ST, McKenzie AN (2012) Type 2 innate lymphoid cells: new players in asthma and allergy. Curr Opin Immunol 24: 707–712. 22985480 10.1016/j.coi.2012.08.009

[17] Witte E, Witte K, Warszawska K, Sabat R, Wolk K (2010) Interleukin-22: a cytokine produced by T, NK and NKT cell subsets, with importance in the innate immune defense and tissue protection. Cytokine Growth Factor Rev 21: 365–379. 20870448 10.1016/j.cytogfr.2010.08.002

[18] Zenewicz LA, Flavell RA (2011) Recent advances in IL-22 biology. Int Immunol 23: 159–163. 21393631 10.1093/intimm/dxr001

[19] Simonian PL, Wehrmann F, Roark CL, Born WK, O’Brien RL, et al (2010) gammadelta T cells protect against lung fibrosis via IL-22. J Exp Med 207: 2239–2253. 20855496 10.1084/jem.20100061 PMC2947077

[20] Bonneville M, O’Brien RL, Born WK (2010) Gammadelta T cell effector functions: a blend of innate programming and acquired plasticity. Nat Rev Immunol 10: 467–478. 20539306 10.1038/nri2781

[21] Goto M, Murakawa M, Kadoshima-Yamaoka K, Tanaka Y, Nagahira K, et al (2009) Murine NKT cells produce Th17 cytokine interleukin-22. Cell Immunol 254: 81–84. 19010461 10.1016/j.cellimm.2008.10.002

[22] Pociask DA, Scheller EV, Mandalapu S, McHugh KJ, Enelow RI, et al (2013) IL-22 is essential for lung epithelial repair following influenza infection. Am J Pathol 182: 1286–1296. 23490254 10.1016/j.ajpath.2012.12.007 PMC3620404

[23] Zenewicz LA, Flavell RA (2008) IL-22 and inflammation: leukin’ through a glass onion. Eur J Immunol 38: 3265–3268. 19016525 10.1002/eji.200838655

[24] Laurence A, O’Shea JJ, Watford WT (2008) Interleukin-22: a sheep in wolf’s clothing. Nat Med 14: 247–249. 18323844 10.1038/nm0308-247

[25] Aujla SJ, Chan YR, Zheng M, Fei M, Askew DJ, et al (2008) IL-22 mediates mucosal host defense against Gram-negative bacterial pneumonia. Nat Med 14: 275–281. 18264110 10.1038/nm1710 PMC2901867

[26] Zhao Y, Yang J, Gao YD, Guo W (2010) Th17 immunity in patients with allergic asthma. Int Arch Allergy Immunol 151: 297–307. 19844129 10.1159/000250438

[27] Besnard AG, Sabat R, Dumoutier L, Renauld JC, Willart M, et al (2011) Dual Role of IL-22 in allergic airway inflammation and its cross-talk with IL-17A. Am J Respir Crit Care Med 183: 1153–1163. 21297073 10.1164/rccm.201008-1383OC

[28] Taube C, Tertilt C, Gyulveszi G, Dehzad N, Kreymborg K, et al (2011) IL-22 is produced by innate lymphoid cells and limits inflammation in allergic airway disease. PLoS One 6: e21799. 21789181 10.1371/journal.pone.0021799 PMC3138740

[29] Nakagome K, Imamura M, Kawahata K, Harada H, Okunishi K, et al (2011) High expression of IL-22 suppresses antigen-induced immune responses and eosinophilic airway inflammation via an IL-10-associated mechanism. J Immunol 187: 5077–5089. 21998459 10.4049/jimmunol.1001560

[30] Zhu Z, Homer RJ, Wang Z, Chen Q, Geba GP, et al (1999) Pulmonary expression of interleukin-13 causes inflammation, mucus hypersecretion, subepithelial fibrosis, physiologic abnormalities, and eotaxin production. J Clin Invest 103: 779–788. 10079098 10.1172/JCI5909 PMC408149

[31] Tichelaar JW, Lu W, Whitsett JA (2000) Conditional expression of fibroblast growth factor-7 in the developing and mature lung. J Biol Chem 275: 11858–11864. 10766812 10.1074/jbc.275.16.11858

[32] Akeson AL, Greenberg JM, Cameron JE, Thompson FY, Brooks SK, et al (2003) Temporal and spatial regulation of VEGF-A controls vascular patterning in the embryonic lung. Dev Biol 264: 443–455. 14651929 10.1016/j.ydbio.2003.09.004

[33] Zhu Z, Ma B, Homer RJ, Zheng T, Elias JA (2001) Use of the tetracycline-controlled transcriptional silencer (tTS) to eliminate transgene leak in inducible overexpression transgenic mice. J Biol Chem 276: 25222–25229. 11331286 10.1074/jbc.M101512200

[34] Zhu Z, Oh MH, Yu J, Liu YJ, Zheng T (2011) The Role of TSLP in IL-13-induced atopic march. Sci Rep 1: 23. 22355542 10.1038/srep00023 PMC3251897

[35] Oh SY, Zheng T, Kim YK, Cohn L, Homer RJ, et al (2009) A critical role of SHP-1 in regulation of type 2 inflammation in the lung. Am J Respir Cell Mol Biol 40: 568–574. 18952567 10.1165/rcmb.2008-0225OC PMC2677436

[36] Park O, Wang H, Weng H, Feigenbaum L, Li H, et al (2011) In vivo consequences of liver-specific interleukin-22 expression in mice: Implications for human liver disease progression. Hepatology 54: 252–261. 21465510 10.1002/hep.24339 PMC3125432

[37] Wolk K, Haugen HS, Xu W, Witte E, Waggie K, et al (2009) IL-22 and IL-20 are key mediators of the epidermal alterations in psoriasis while IL-17 and IFN-gamma are not. J Mol Med (Berl) 87: 523–536. 19330474 10.1007/s00109-009-0457-0

[38] Hellings PW, Kasran A, Liu Z, Vandekerckhove P, Wuyts A, et al (2003) Interleukin-17 orchestrates the granulocyte influx into airways after allergen inhalation in a mouse model of allergic asthma. Am J Respir Cell Mol Biol 28: 42–50. 12495931 10.1165/rcmb.4832

[39] Dumoutier L, Van Roost E, Ameye G, Michaux L, Renauld JC (2000) IL-TIF/IL-22: genomic organization and mapping of the human and mouse genes. Genes Immun 1: 488–494. 11197690 10.1038/sj.gene.6363716

[40] Liang M, Wang J, Chu H, Zhu X, He H, et al (2013) Interleukin-22 inhibits bleomycin-induced pulmonary fibrosis. Mediators Inflamm 2013: 209179. 23476100 10.1155/2013/209179 PMC3588191

[41] Kim KW, Kim HR, Park JY, Park JS, Oh HJ, et al (2012) Interleukin-22 promotes osteoclastogenesis in rheumatoid arthritis through induction of RANKL in human synovial fibroblasts. Arthritis Rheum 64: 1015–1023. 22034096 10.1002/art.33446

[42] Cobleigh MA, Robek MD (2013) Protective and pathological properties of IL-22 in liver disease: implications for viral hepatitis. Am J Pathol 182: 21–28. 23159948 10.1016/j.ajpath.2012.08.043

[43] Res PC, Piskin G, de Boer OJ, van der Loos CM, Teeling P, et al (2010) Overrepresentation of IL-17A and IL-22 producing CD8 T cells in lesional skin suggests their involvement in the pathogenesis of psoriasis. PLoS One 5: e14108. 21124836 10.1371/journal.pone.0014108 PMC2991333

[44] Nograles KE, Zaba LC, Shemer A, Fuentes-Duculan J, Cardinale I, et al.. (2009) IL-22-producing “T22” T cells account for upregulated IL-22 in atopic dermatitis despite reduced IL-17-producing TH17 T cells. J Allergy Clin Immunol 123: 1244–1252 e1242. 10.1016/j.jaci.2009.03.041 PMC2874584 19439349

[45] Sugimoto K, Ogawa A, Mizoguchi E, Shimomura Y, Andoh A, et al (2008) IL-22 ameliorates intestinal inflammation in a mouse model of ulcerative colitis. J Clin Invest 118: 534–544. 18172556 10.1172/JCI33194 PMC2157567

[46] Takahashi K, Hirose K, Kawashima S, Niwa Y, Wakashin H, et al.. (2011) IL-22 attenuates IL-25 production by lung epithelial cells and inhibits antigen-induced eosinophilic airway inflammation. J Allergy Clin Immunol 128: 1067–1076 e1061–1066. 10.1016/j.jaci.2011.06.018 21794904

[47] Lecart S, Morel F, Noraz N, Pene J, Garcia M, et al (2002) IL-22, in contrast to IL-10, does not induce Ig production, due to absence of a functional IL-22 receptor on activated human B cells. Int Immunol 14: 1351–1356. 12407026 10.1093/intimm/dxf096

