# Supplementary figures and images for: Immune Modulatory Effects of IL-22 on Allergen-Induced Pulmonary Inflammation
Source: PLoS One. 2014 Sep 25;9(9):e107454. doi: 10.1371/journal.pone.0107454 (PMC4177833; doi:10.1371/journal.pone.0107454)

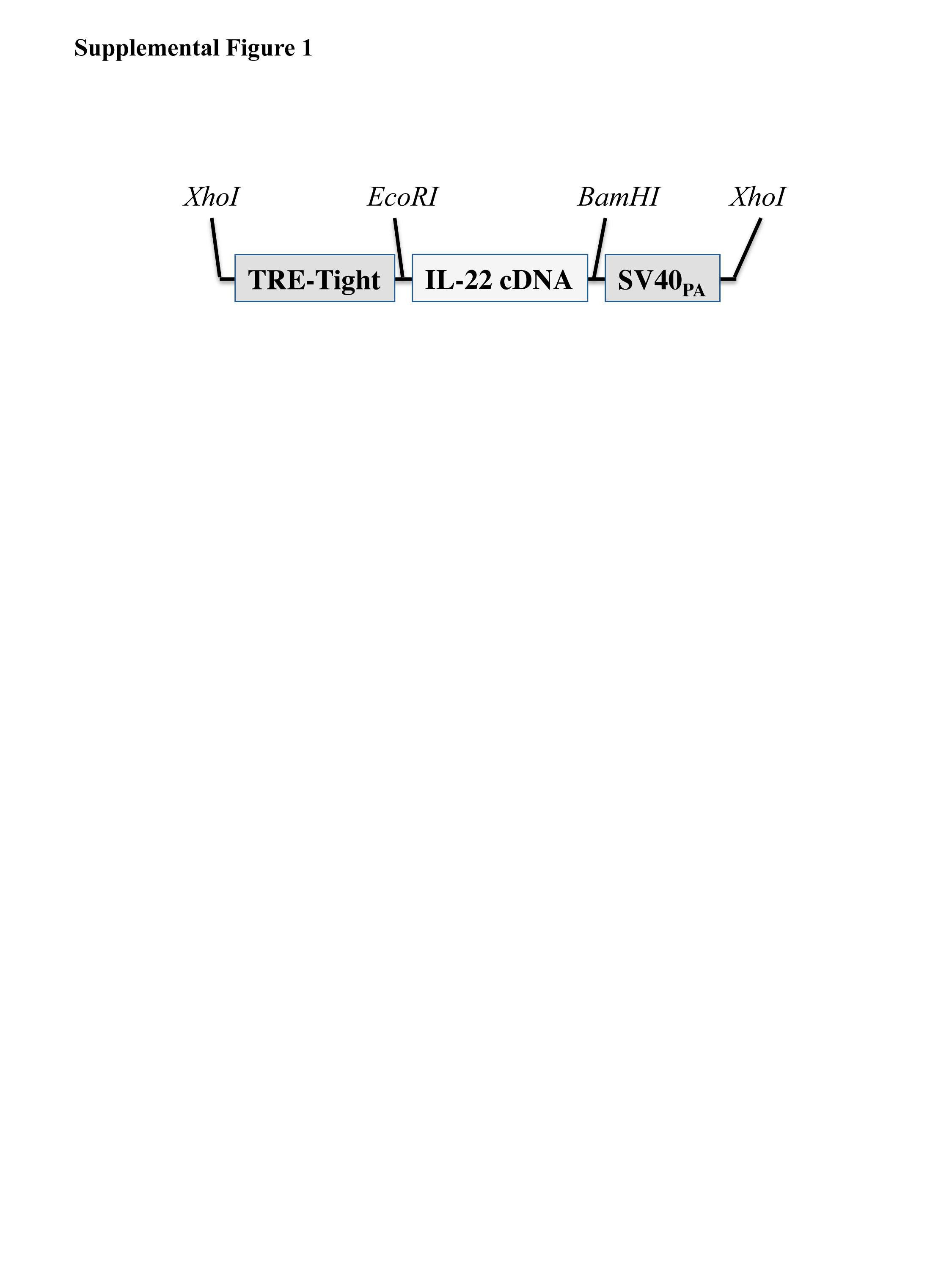

Supplement: Figure S1 — Schematic DNA construct of TRE-Tight-IL-22 transgene. IL-22 cDNA was inserted into the multiple cloning site (MCS) of pTRE-Tight vector (Clontech) using restriction enzymes and microinjected into fertilized eggs as described. (TIF) [file pone.0107454.s001.tif]

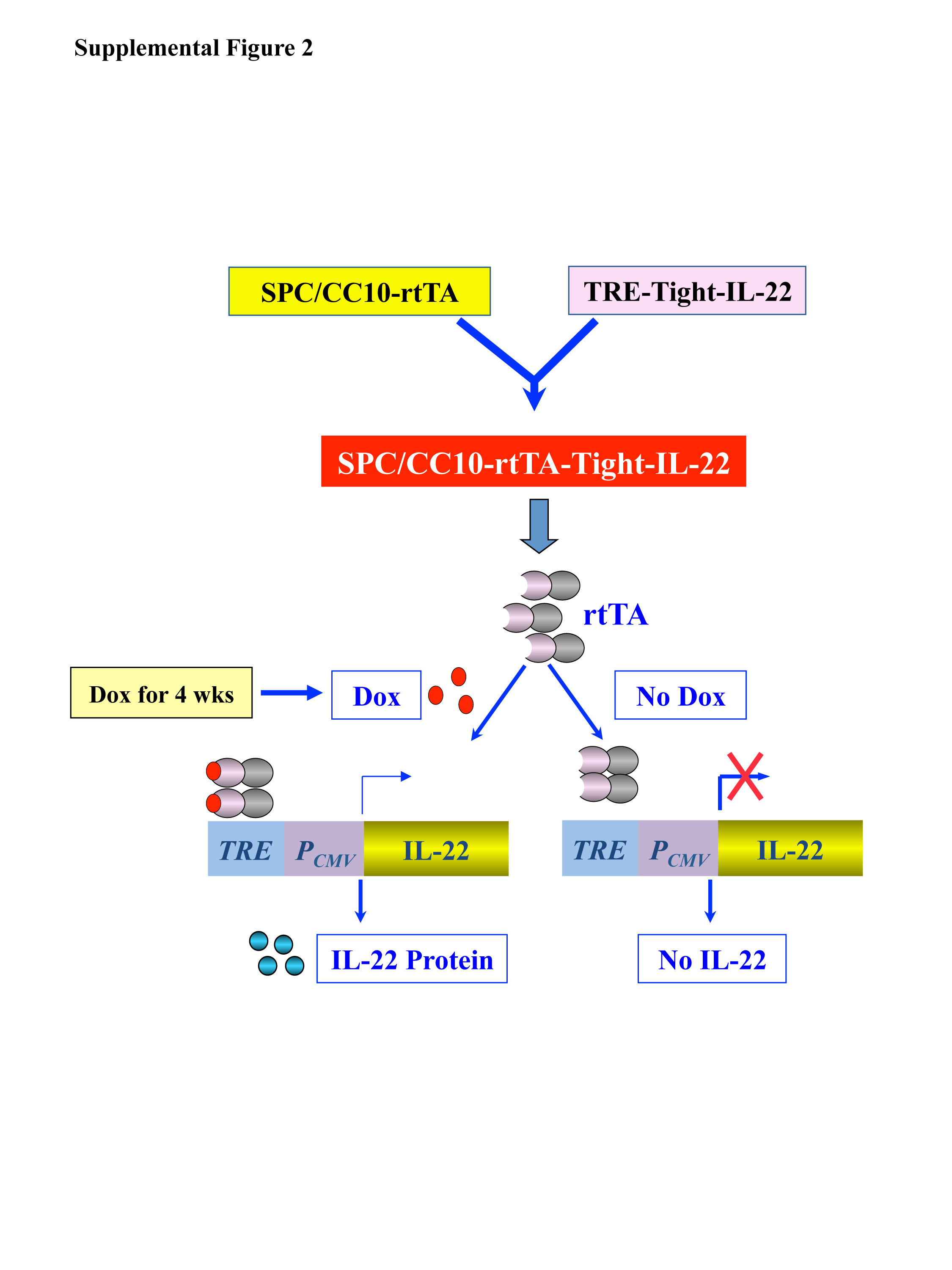

Supplement: Figure S2 — Generation of SPC- or CC10-rtTA-TRE-Tight-IL-22 (also called SPC- or CC10-IL-22) mice. As illustrated, SPC-rtTA or CC10-rtTA mice were crossbred with TRE-Tight-IL-22 mice to obtain SPC- or CC10-IL-22 double positive mice. The IL-22 transgene was activated by doxycycline (Dox) in the drinking water for 4 weeks. ELISA, Western blot, immunohistochemistry (IHC) and immunofluorescence (IF) were performed to identify the expression of IL-22 in the lung. Without Dox, no IL-22 was detected in the BAL or in the lung. (TIF) [file pone.0107454.s002.tif]
