# Supplementary material for: Immune Modulatory Effects of IL-22 on Allergen-Induced Pulmonary Inflammation
Source: PLoS One. 2014 Sep 25;9(9):e107454. doi: 10.1371/journal.pone.0107454 (PMC4177833; doi:10.1371/journal.pone.0107454)
Supplement: File S1 — (DOCX) [file pone.0107454.s003.docx]

***Supporting Information***

**Immune Modulatory Effects of IL-22 on Allergen-Induced Pulmonary Inflammation**

Ping Fang, MD, PhD^1, 2^, Li Zhou, PhD^2^, Yuqi Zhou, MD, PhD^2^, Jay Kolls, MD^3^,

Tao Zheng, MD^2^, and Zhou Zhu, MD, PhD^2^

^1^Respiratory Department

The Second Affiliated Hospital

Xi’an Jiaotong University School of Medicine

157 Xiwu Road,

Xi’an, Shaanxi, China 710004

^2^Division of Allergy and Clinical Immunology

Department of Internal Medicine

Johns Hopkins University School of Medicine

5501 Hopkins Bayview Circle, 1A2

Baltimore, MD 21224

^3^Division of Pediatric Rheumatology

Children’s Hospital of Pittsburgh

University of Pittsburgh School of Medicine

Pittsburgh, PA 15224

**Correspondence:**

Zhou Zhu, MD, PhD

Email: [zhou.zhu@yale.edu](mailto:zhou.zhu@yale.edu)

Current address:

Zhou Zhu and Tao Zheng, Yale University School of Medicine

Li Zhou, Wuhan University School of Medicine

Yuqi Zhou, Zhongshan School of Medicine, SYSU

**Materials and Methods**

*Generation of lung-specific inducible IL-22 transgenic mice*

Transgenic mice on C57BL/6 genetic background carrying the transgene TRE-Tight-IL-22 were generated as the following. Mouse IL-22 cDNA was PCR amplified from a plasmid containing mouse IL-22 using primers: 5’-GCG-AAT-TCC-CCC-TTC-ACC-GC-3’ and 5’-CGC-GGA-TCC-TTC-CAG-TTT-AAT-3’ with *Eco*RI and *Bam*HI sites. After restriction enzyme digestion, the IL-22 cDNA fragment was inserted into the multiple cloning site of the pTRE-Tight vector (Clontech). The DNA fragment containing the TRE-Tight promoter, IL-22 cDNA, and the SV40 polyadenylation signal sequence was excised with *Xho*I, purified, and microinjected into pronuclei as described previously [[1](#_ENREF_1)]. To obtain mice that can express IL-22 specifically and inducibly in the lung, TRE-Tight-IL-22 mice were crossbred with CC10-rtTA or SPC-rtTA transgenic mice (kindly provided by Dr. Jeffrey Whitsett from the University of Cincinnati) to produce double transgenic CC10-rtTA-IL-22 or SPC-rtTA-IL-22 Tg(+) mice. The breeding also produced single transgenic mice, which were used for further breeding and transgenic negative mice, which were used as Tg(-) littermate controls in the experiments. The genotypes of the mice were determined by PCR using specific primers for CC10, SPC and TRE-Tight-IL-22.

*Histology and immunohistochemistry (IHC)*

Hematoxylin and eosin and Alcian blue (AB) stains were performed on lung sections after ﬁxation with neutral buffered formalin at 4°C overnight, embedded in paraffin, sectioned at 5 μm for histological analysis as described previously [[2](#_ENREF_2)]. For immunohistochemistry experiments, after the sectioned tissues were rehydrated, endogenous peroxidase was quenched by 1% hydrogen peroxide diluted in methanol for 7 minutes in room temperature. After pre-blocking with blocking serum (donkey serum) for 30 minutes, a rat anti-mouse major basic protein (MBP) monoclonal antibody (a kind gift from Drs. Nancy and James J. Lee, Mayo Clinic, Scottsdale, AZ) was applied at 1:500 dilution to stain eosinophils. Similarly, for IL-22 positive cells, goat anti-mouse IL-22 monoclonal antibody (Santa Cruz Biotechnology Inc., Santa Cruz, CA) was applied at a 1:180 dilution. Appropriate ABC staining systems were used to visualize the target proteins in the tissues (Santa Cruz Biotechnology).

*Immunoﬂuorescence*

Immunoﬂuorescence was performed on deparafﬁnized mouse lung tissue slides. Antigen unmasking was performed by put deparafﬁnized slides in 10 mM sodium citrate buffer pH 6.0, then maintain at a sub-boiling temperature for 10 minutes. Slides were cooled for 30 minutes and then incubated in ice-cold 100% methanol for 10 minutes at –20°C. These slides were then blocked with donkey blocking solution of 10% donkey serum (Sigma-Aldrich, St. Louis, MO), 1% BSA, and 0.5% Tween 20 in PBS for 1 hour at RT. After washing, tissue sections were incubated at 4°C overnight with rabbit anti mouse phospho-Stat3 (Tyr705) (Cell Signaling, Danvers, MA). After rinse, tissue sections were incubated with Alexa Fluor 488-labeled donkey anti-rabbit IgG (A10039; Invitrogen) and DAPI (Roche Diagnostics, Mannheim, Germany) at RT for 2 hours. Finally, tissue sections were mounted using PermaFluor (Thermo Fisher Scientiﬁc) and examined using a Zeiss LSM 510 laser scanning confocal microscope (Carl Zeiss) at 350 nm to assess p-STAT3 and 405 nm to assess cell nuclei.

*Analysis of mRNA*

Total cellular RNA from lung tissue was obtained using Trizol reagent (Invitrogen, Carlsbad, CA). Reverse transcription was performed using 0.5 μg total RNA for first-strand cDNA synthesis with SuperScript II RNase H^-^ Reverse Transcriptase (Invitrogen) in a total volume of 20 μl. One μl resulting reverse-transcription product was used for PCR amplification. PCR conditions to amplify specific genes were 95°C for 4 minutes for initial denaturing followed by 30 cycles of 94°C for 1 minute, 60°C for 1minute, and 72°C for 1 minute. The mRNA of IL-22 was evaluated using speciﬁc primers (sense primer 5’-GCG-AAT-TCC-CCC-TTC-ACC-GC-3’, anti-sense primer 5’-CGC-GGA-TCC-TTC-CAG-TTT-AAT-3’). The mRNA of β-actin was used as an internal reference (sense primer 5’-GTG-GGC-CGC-TCT-AGG-CAC-CAA-3’, anti-sense primer 5’-CTC-TTT-GAT-GTC-ACG-CAC-GAT-TTC-3’).

**Figure S1**. Schematic DNA construct of TRE-Tight-IL-22 transgene. IL-22 cDNA was inserted into the multiple cloning site (MCS) of pTRE-Tight vector (Clontech) using restriction enzymes and microinjected into fertilized eggs as described.

**Figure S2**. Generation of SPC- or CC10-rtTA-TRE-Tight-IL-22 (also called SPC- or CC10-IL-22) mice. As illustrated, SPC-rtTA or CC10-rtTA mice were crossbred with TRE-Tight-IL-22 mice to obtain SPC- or CC10-IL-22 double positive mice. The IL-22 transgene was activated by doxycycline (Dox) in the drinking water for 4 weeks. ELISA, Western blot, immunohistochemistry (IHC) and immunofluorescence (IF) were performed to identify the expression of IL-22 in the lung. Without Dox, no IL-22 was detected in the BAL or in the lung.

**Reference**

1. Zhu Z, Homer RJ, Wang Z, Chen Q, Geba GP, et al. (1999) Pulmonary expression of interleukin-13 causes inflammation, mucus hypersecretion, subepithelial fibrosis, physiologic abnormalities, and eotaxin production. J Clin Invest 103: 779-788.

2. Zheng T, Zhu Z, Wang Z, Homer RJ, Ma B, et al. (2000) Inducible targeting of IL-13 to the adult lung causes matrix metalloproteinase- and cathepsin-dependent emphysema. J Clin Invest 106: 1081-1093.
